# An insight into the experience of healthcare providers and users of women-friendly health services in Jordan—a mixed method study

**DOI:** 10.3389/fgwh.2026.1772412

**Published:** 2026-04-08

**Authors:** Eman Zmaily Dahmash, Ali Al-Gharabli, Thaira Al-Madi, Rahma Jebreel, Ahmad Shatat, Worood Al khob, Reem Al-Reyati, Wafaa Shariedeh, Walaa Shqerat, Ghada Alsaad

**Affiliations:** 1 Faculty of Pharmacy, Isra University, Amman, Jordan; 2 Department of Chemical and Pharmaceutical Sciences, School of Life Sciences, Pharmacy and Chemistry, Kingston University London, Kingston upon Thames, United Kingdom; 3 Sexual Reproductive Health Programme, UNFPA in Jordan, Amman, Jordan; 4 Department of Accreditation, Healthcare Accreditation Council, Amman, Jordan; 5 Health Care Department, ational Woman’s Health Care Centre, Amman, Jordan; 6 Directorate of Professional Training and Planning, Jordanian Royal Medical Services, Amman, Jordan; 7 Directorate of Nursing and Allied Medical Profession, Jordanian Royal Medical Services, Amman, Jordan; 8 Studies and Planning Department, National Woman's Health Care Centre, Amman, Jordan

**Keywords:** integrated services, Jordan, low-middle-income countries, primary healthcare, satisfaction, women friendly health services

## Abstract

**Background:**

Women's Friendly Health Services (WFHS) are designed to be available, accessible, and affordable for women, providing care throughout their lives from youth to postmenopausal stages. These integrated services focus on women's needs, follow evidence-based practices, and are provided by a qualified and trained team. In 2022, Jordan took the initiative and implemented a WFHS in 9 primary health centres aiming at improving healthcare services for women across the life. However, there is limited data on WFHS in Jordan. Therefore, the aim of this study was to investigate the experiences of healthcare providers (HCP) and users with women-friendly healthcare services in Jordan and to explore the challenges encountered in implementing this initiative.

**Methods:**

This study involved a qualitative approach utilising semi-structured focus group discussions with 18 HCPs at piloted WFHS providing centres. Additionally, a quantitative was conducted with women utilising the WFHS in the piloted centres using a questionnaire.

**Results:**

The analysis of the focus group discussions revealed key themes with several subthemes: high staff turnover, limited resources, and the need for additional integrated services. A survey of 393 women across 9 pilot centres showed generally positive perceptions of WFHS with high scores for respect and dignity (86%) and overall service quality (81.4%). The questionnaire was highly reliable (Cronbach alpha = 0.915). Nevertheless, women identified issues with staff greetings, and the lack of designated breastfeeding and diaper changing areas. WFHS was well-perceived by women, with discrimination-free care scoring the highest at 4.65 ± 0.592 (93%) and timely care scoring the lowest at 3.44 ± 1.125 (68.8%). Additionally, waiting time was identified as the main drawback to WFHS, scoring the lowest among all WFHS-friendly care-related questions at 3.16 ± 1.354 (63.2%).

**Conclusions:**

The findings from the two methods aligned, revealing that HCP and women shared similar concerns. While the WFHS were well-received by participants and is seen as a positive initiative, further efforts are required to enhance and scale this initiative across Jordan and other Low-Middle-Income Countries (LMICs). Offering integrated and respectful care can improve healthcare utilisation and reflect high-quality service delivery.

## 1 Introduction

Despite advancements in the health system, particularly in women's health, global statistics continue to tell a disturbing story. Globally, around 800 women die daily from pregnancy and childbirth complications, with 1 in 3 women globally experiencing violence. Additionally, depression is twice as common among women than men (1). The World Health Organisation (WHO) has reported that women and girls continue to face health challenges due to inequalities and disrupted access to vital health and support services (2). In 2021, WHO set six priorities for women and reported that: “Concrete action is needed to ensure women and girls in all their diversity can enjoy the right to health—during, and beyond the COVID-19 pandemic” (1). The United Nations Population Fund (UNFPA) has also reported similar challenges faced by women and girls globally, citing the significant impact of gender inequalities and disruptions to essential health services on their health and well-being (3). Despite efforts in Jordan to improve women's health, more work needs to be done. While there has been a reduction in maternal mortality in Jordan over the past 20 years, with numbers dropping from 64 in 100,000 in 2000 to 41 in 100,000 in 2020, the current numbers are still alarming (4).

Sexual and reproductive health (SRH) reflects the overall well-being of societies and countries. It goes beyond maternal and child health. The Millennium Development Goals and Sustainable Development Goals 3 and 5 emphasise SRH aspects and indicators integrated into relevant international and regional strategies (5). Several studies and reports on SRH in Jordan have highlighted challenges in achieving SRH targets, including limited integration of SRH programs in primary healthcare, a lack of programs to assess SRH services, and user satisfaction (6).

Furthermore, recent reviews related to cervical cancer screening in Jordan (7) and breast cancer screening (8), reported that some of the main obstacles to efficient screening included a lack of accessible and available high-quality services, the scarcity of comfort and privacy in healthcare centres, and a fear of cancer diagnosis (9). Therefore, there is an urgent need for accessible, affordable, and comprehensive women's healthcare services at the primary level in Jordan. As structured primary care programs and services are lacking in Jordan, following patients with cancer who have treatment-related complications or even comorbidities while undergoing anticancer therapy and beyond puts a lot of pressure on cancer centres to provide such service (8).

WHO country cooperation strategic health agenda for Jordan (2018–2019) stated a strategic priority that entailed “Promoting Health Across the Life—Course” and set a focus area for WHO cooperation. It included an outcome: increased access to interventions for improving the health of women, newborns, children and adolescents (10). Women-friendly health services (WFHS) is an approach to healthcare giving to create an enabling environment at all levels of women's healthcare services to improve women's access to safe health services (11, 12). The global understanding of women's health extends beyond maternity; it is sought as the provision of care throughout the life cycle of women (youth, maternity, postnatal and postmenopausal)—in other words, from birth to death (13, 14).

UNFPA plays a vital role in this strategic focus by assisting in the delivery of comprehensive sexual and reproductive health services, particularly for women and adolescents. UNFPA's activities prioritize the provision of family planning, skilled birth attendance, and emergency obstetric care. These efforts play a crucial role in reducing maternal mortality and morbidity. Additionally, UNFPA collaborates with local partners to promote youth-friendly services and comprehensive sexuality education, addressing the specific needs of young people and adolescents. By advocating for policies that support women's health across their life course and providing technical assistance and capacity-building to healthcare providers, UNFPA reinforces WHO's strategic goals and enhances the overall health and well-being of women in Jordan (15, 16).

Women's Friendly Health Services (WFHS) are healthcare services provided in an integrated manner, focusing on the needs and expectations of women in various stages of their lives, and taking into consideration the characteristics of different groups and societies (17). The services include (a) Sexual and Reproductive Health (SRH) Services that cover antenatal and postnatal healthcare, family planning, obstetric care, cancer screening, menopause and infertility care, (b)_ Gender-Based Violence (GBV) Response, (c) as well as Integrated Care and Accessibility (18). It is expected that WFHS follow evidence-based clinical practices, understand local challenges, and be provided through a qualified and trained team that women can trust to take care of their health needs, including medical, psychological, and social needs (17). UNFPA's work in Jordan underscores the need for continued efforts to address these challenges through comprehensive health services, policy changes, and targeted interventions to support women and girls, particularly in vulnerable and crisis-affected settings (19).

Women-Friendly Health Services (WFHS) are designed to be accessible, affordable, and located close to women's communities. They provide safe, effective, and high-quality health services and maternal care. Within the WFHS model, Healthcare providers are encouraged to engage in facility-level decision-making, which strengthens their capacity to respond effectively to women's needs. WFHS also empower users by respecting their rights to information, choice, safety, privacy, and dignity, while remaining sensitive to cultural and social norms, ensuring care is both effective and women-centred (20).

By the end of 2024, women made up 50.1% of Jordan's population, with 60.7% of working age (15–64 years) and 52.5% of reproductive age (15–49 years). Educational indicators have improved: female literacy reached 93.2% (21). However, higher education has not translated into comparable workforce participation or social autonomy, as female labor force involvement remains low (14.6%). Socioeconomic disparities persist, especially among women in rural, underserved, and refugee communities, limiting access to services and health literacy. In Jordan, traditional gender norms and male-dominated decision-making further restrict women's autonomy in healthcare (22). These factors underscore the need for women-friendly health services that provide equitable, respectful, and accessible care for diverse groups, including adolescents, mothers, and postmenopausal women.

Jordan healthcare system is based on public, private and non-governmental (NGO) services. The public services include the Ministry of Health and Royal Medical Services, which provide care to about 60%–70% of the population. Consequently, WFHS are essential to provide equitable, culturally sensitive, and accessible care—covering reproductive, adolescent, and postmenopausal health—that is not universally available under the standard system (23).

In response to the call from WHO and UNFPA to develop high-quality women's health-related programs, the National Women's Health Care Centre (NWHCC) created the initiative of WFHS in 2018 as part of the centre's vision towards a healthier woman in Jordan. Accordingly, in collaboration with UNFPA in Jordan and the Health Care Accreditation Council (HCAC), the NWHCC developed “Women-Friendly Health Service Standards” with an aim to identify standards that are valued by women and consist of the best evidence-based practices to scale up women's healthcare in Jordan. NWHCC developed standards for women-friendly healthcare services to be applied in different primary and comprehensive healthcare centres in Jordan. Also, NWHCC developed standard operating procedures (SOPs) related to women's health to support the provision of high-quality, equitable reproductive health services for women throughout their life cycle.

The standards embraced the key principles of women-friendly services that encompass: (a) health is a right for all women of any age; (b) women's participation is important in all stages of care; (c) care is comprehensive in nature; (d) a complete and responsible referral system is in place; (e) a holistic approach to care that continues throughout the life course is implemented; and (f) access to all women without discrimination is available (12).

The Women Friendly Healthcare Centres (WFHC) program that was initiated by NWHCC through UNFPA support, strengthens women-centred services at primary healthcare centres. Since 2021, HCAC has implemented the program, conducting situation analyses, updating standards and SOPs in line with national and international guidelines, supporting WFHS implementation, and establishing a survey-based recognition system to assess centres’ compliance with WFHS standards.

The standards were piloted in nine of the ten healthcare centres across Jordan in 2022 (one centre of the ten piloted was not operating due to renovation). To address the geographic distribution of the WFHS, the pilot centres were distributed across the three main regions of Jordan to ensure national representation. Specifically, three centres were in the central region, two in the northern region, and four in the southern region. The administrative oversight of the participating centres was distributed across three health system sectors: three centres operated under the Ministry of Health, three under the Royal Medical Services (Military Medical Services), and three under non-governmental organisations (NGOs). The centres provide services to registered clients free of charge or at minimal cost, thereby promoting equitable access to care.

WFHS initiative was assessed using two parallel convergent mixed method research design. The first phase involved focus group discussions with key healthcare providers from the piloted PHC. This phase adopted a qualitative approach to delve into their in-depth experience related to the initiative. The second phase employed a quantitative approach, focusing on a cross-sectional study targeting women using the WFHS services within the piloted centres. An assessment tool was used based on the principles of the WFHS and WHO recommendations. The purpose of this phase was to gather data capturing the experiences and views of women regarding WFHS, aiming to gain a wide, diverse perspective and represent the views of as many women as possible. This data would be used in key decision-making conversations across Jordan.

This research provided valuable insights into the existing gaps and challenges in successfully implementing WFHS programs and standards. Qualitative methods, semi-structured focus groups were used to uncover the complexities, challenges, and gaps in WFHS programs and standards.

For the second phase, ensuring an effective and sustainable WFHS program depends on understanding the factors that impact women's satisfaction and perception of the program. A survey was conducted to gather data on women's experiences, views, and perceptions of WFHS.

This study aimed to examine Jordan's WFHS program by exploring women's experiences and concerns at piloted healthcare centres, alongside healthcare providers’ perspectives on the program's added value and implementation challenges, to generate a concise, integrated understanding of WFHS effectiveness and opportunities to enhance women-centred care across Jordan.

## 2 Methodology

### 2.1 Phase 1: focus group

Focus groups with healthcare providers (HCP) from the piloted healthcare centres that employed the WFHS standards, and services were conducted to explore challenges, gaps and priorities in the WFHS program, as well as barriers to accessing and provision of the services. Focus group methods were chosen as they can provide rich qualitative data rapidly, enabling exchanges of views between participants, as well as being a useful method for conducting prioritisation exercises (24).

#### 2.1.1 Study participants

Healthcare providers who work in all the WFHS-piloted PHCC were invited to participate through formal invitation letters via the Ministry of Health. The HCPs were PHCC doctors, nurses, midwives, or nurses/quality coordinators. A total of 2 sessions were conducted, and each focus group was comprised of nine HCPs. Informed consents were obtained from the participants before the focus group took place. Participants received information on the project which was sent with the invitation letter. Participation was obtained through formal letter to the centres. Given the qualitative and exploratory nature of the section of the research, which aimed to understand healthcare providers’ perceptions regarding the WFHS, convenient sampling was considered appropriate.

#### 2.1.2 Inclusion and exclusion criteria

Inclusion criteria for study participation were HCPs, defined as being working as a doctor, nurse, midwife, or nurse/ quality coordinator working in one of the piloted PHCCs that implemented the WFHS standards. The exclusion criteria were (a) non-HCPs; (b) eligible HCPs not willing to participate; and (c) HCPs from the piloted PHCC that recently joined the work within the last three months.

#### 2.1.3 Semi-structured focus group methodology

The participants were distributed in the focus group sessions on two separate occasions. The duration of each session was 60 min. All focus group sessions took place online using the Zoom online application. All sessions were digitally recorded for analysis.

Focus groups were conducted using a structured guide developed by the research team, including four main questions and follow-up probes to explore participants’ experiences with WFHS. Participants were recruited from different piloted centres. Overall, the focus group directed on opening conversations around the following questions:

1. What are the most important healthcare issues for women that should be incorporated in the WFHS?
2. Thinking about WFHS program and the topics we have discussed, do you think there are barriers to healthcare providers in providing such a comprehensive service? [PROMPT] What are those barriers/challenges?”
3. What are the additional issues that need to be addressed to improve the WFHS program within your centre and in Jordan?
4. What might help to have a more effective WFHS program?” & “Can you think of any good examples where the program can be best used or improved?”

Sessions were moderated by a trained researcher in health services research and an expert in moderating discussion groups. One moderator led the sessions. At the beginning of each session, the moderator introduced the topic of the session and clarified the purpose of this study. Next, the moderator facilitated the discussion (25). The focus group guide included the above 4 main questions about WFHS, with PROMPT to explore responses in depth. Probed items were part of the component list and the moderator geared the discussion towards the targeted questions. The questions were previously sent to the participant in the invitation letters.

#### 2.1.4 Data analysis

Immediately after each session, the recording was transcribed and analysed by two researchers, thus providing the opportunity to detect gaps, reveal unclear comments, and facilitate the rolling interview guide for the upcoming session of the focus groups. Two reviewers independently coded all statements of the participants’ concerning components of focus group questions. For detailed textual analysis of the data Nvivo software (QSR international version 14.23.0). Two reviewers independently attempted to unveil trends in the statements and relations between components, to cluster the components into a small number of domains, and to construct an overall statement for key recommendations. An inductive approach was primarily used in the thematic analysis, allowing themes to emerge directly from participants’ responses, while some deductive coding was applied based on the WFHS program standards (26, 27).

### 2.2 Phase 2: cross-sectional study using a survey questionnaire (women-friendly services survey questionnaire)

#### 2.2.1 Study design

An institutional-based cross-sectional study was conducted at the piloted healthcare centres in Jordan, between November 2023 and February 2024. The study was conducted through one–on–one surveys to capture the experiences and views of women, gaining a wide and diverse perspective to represent the views of as many women as possible who used the WFHS within the piloted healthcare centres.

#### 2.2.2 Study participants

All women who attend the piloted healthcare centres that provide WFHS in Jordan are the source population. Women who received services within the bundle of WFHS were included.

#### 2.2.3 Sample size and procedure

Eligible participants attending the clinic waiting areas were invited to participate in the study. The participants were invited to participate in this study through a trained data collector attending the clinic on designated days. Women were approached in person by the data collector and asked to voluntarily participate in the study. All accepted and eligible women were given a link to fill out the questionnaire. All participants voluntarily participated in the study and were thus considered exempt from written informed consent The study aims and objectives were clearly explained at the beginning of the survey. The target sample size was estimated based on WHO recommendations for the minimal sample size needed for population-based studies (28). Using a confidence interval of 95%, a standard deviation of 0.5, and a margin of error of 5%, the required sample size was 385 participants from each study population. Data collectors were trained on the objective, method, sampling technique, ethical issues, data collection instrument, and data collection procedure before data collection. A total of 393 women completed the questionnaire, yielding a response rate that exceeded 87%.

#### 2.2.4 Inclusion and exclusion criteria

The inclusion criteria are as follows: participants were women (a) living currently in Jordan; (b) had no apparent cognitive deficit; (c) received healthcare services in one of the piloted PHCCs that provided WFHS. Participants were excluded if they were as follows: (a) below 14 years of age; (b) unable to understand Arabic language; (c) unable to participate due to physical or emotional distress and (d) participants who were coming to the centre for the first time.

#### 2.2.5 Data collection tool

A structured questionnaire which was adapted from different works of literature (29, 30), and consisted of three sections. The first section was used to assess socio-demographic characteristics of the women with 10 items, the second section was used to assess facilities services use and providers-related characteristics with 19 items, and the third section was used to measure the level of women-friendly care provision experienced by women during their visit to the centres using 16 verified questions. In this study, 2 data collectors and 3 key researchers were assigned to facilitate the data collection. The English version of the questionnaire was translated into Arabic (local language) to obtain data from the study participants and to ensure clarity of its content. Back translation was used to ensure accuracy and consistency.

#### 2.2.6 Study variables, outcome variable, and operational definitions

Socio-demographic, and facility-related, variables are study variables whereas the level of women-friendly care provision is the outcome variable. A series of questions related to the level of women-friendly care provision will be asked. A 5-point Likert scale scores were used and values of at least mean and above were considered as a good level of women-friendly care provision. Study participants who scored above the average score towards women-friendly care provision questions were categorised as getting a "good level of women-friendly provision" and else were categorised as getting poor women-friendly care provision. In this study, women's satisfaction and ‘quality of care’ are used interchangeably to reflect women's perceptions and experiences. While conceptually distinct in the literature, they were used to align with the questionnaire's focus on users’ evaluation of WFHS.

#### 2.2.7 Ethical approval

Participants were required to sign a consent form that accompanied the invitation letter for the focus groups. In addition, a consent statement was provided at the beginning of the online survey, which explained the purpose of the research and emphasised that participation was voluntary. It was explicitly stated that there were no direct benefits for the participants and that their involvement would not affect their employment or work assessments. As a result, all participants who completed and submitted the survey provided informed consent to be included in the study. The study protocol received approval from relevant authorities, including the Ministry of Health (MoH) with approval number MoH/REC/2023.299 on 16/08/2023, the Royal Medical Services (RMS) with approval number T/F/3/1/1821 on 10/2023, the NWHCC with approval number 1/2/1/1092 on 19/11/2023, and UNFPA on 1/8/2023.

#### 2.2.8 Data analysis and presentation

The data were analysed using SPSS software version 26.0. Descriptive statistics were tabulated for the variables. Correlation and regression analysis were employed to determine the association between the independent variables and the outcome variables.

## 3 Results

### 3.1 Phase one: focus groups

#### 3.1.1 Participant characteristics

We received contributions from the 9 primary healthcare centres (PHC) that were piloted. A total of 18 participants joined in the focus groups. The distribution of the participants according to their profession was 27.8% doctors (D), 22.2% nurses (N), 38.9% quality improvement coordinators [nurses with additional QI roles (QI)], and 11.1% midwives (MW). The centres*’* distribution according to governing body included 3 governed by the Ministry of Health (MoH), 3 were run by the Jordanian Royal Medical Services/ Military (RMS), and 3 were non-governmental centres (NGO). Additionally, 88.9% of the participants were female (F), which is consistent with the actual workforce in primary healthcare services in Jordan, while only 11.1% represented male participants (M).

#### 3.1.2 Codes and thematic analysis

Four main questions were raised during the focus group sessions. The thematic analysis using Nvivo software of the collected data resulted in emerging themes with several subthemes, each of which highlights healthcare providers’ experiences in WFHS (Supplementary Appendix Table S1). The results show that healthcare providers’ have a variety of experiences about WFHS.

##### 3.1.2.1 Domain 1: important healthcare issues for women that should be incorporated into the WFHS

###### 3.1.2.1.1 Theme 1:1 community integration and communication

The HCP discussed several issues that are essential components of WFHS, which were divided into four subthemes. The first subtheme focuses on community-related services to be incorporated into WFHS, particularly those related to providing a community awareness program to explain all specific services related to women-friendly healthcare services that should be integrated with community services. This integration can maximise the benefits of WFHS within Primary Healthcare (PHC) centres.

“One of the key issues we need to address is health awareness. We need to focus on health awareness campaigns”. (N, F)

“We should focus on providing mental and psychological support services that integrate with community services. This approach will help us to reach a larger number of targeted women”. (N, F)

"We have observed several cases of Hepatitis A among young people in the local community. In response, we conducted a series of educational and counselling sessions for schoolteachers, cleaners, and students to raise awareness”. (D, M)

"We must raise awareness and promote the idea that prevention is better than treatment within the community”. (MW,F)

"Women should visit the PHC centres not only for treatment but also for screening and educational purposes. We should promote the concept of a "Healthy Women Clinic”, similar to the "Healthy Child Clinic". This clinic can conduct routine and regular screening activities as well as educational sessions". (D, F)

**Community integration** was further addressed as a vital issue for WFHS as it can reach remote areas and can cover services that are new to PHC:

“We have started reaching out to teenagers and young girls through the school”. (QI, F)

“Women living in remote areas are being reached through community services. We have set up various teams for community health screening and due to accessibility issues to the centre, we have conducted numerous community outreach activities”. (D, F).

"Engaging and involving the school health program in the WFHS program, particularly for young women/teenagers, will help attract this group, which typically does not seek care at the PHC". (N, F)

"We need to involve community services to attract women in menopausal age". (N, F)

To improve the services, **family support** was a key issue that all participants reached a consensus on:

"Based on our experience, we have observed that women within the WFHS require additional support from their family members". (D, F)

"It is important to clearly outline the supportive role of husbands in the guidelines, especially for prenatal and postnatal services. Their support is crucial for the success of these services. We should encourage husbands to participate in counselling sessions for prenatal and postnatal services and parents’ attendance for teenagers’ counselling services". (N, F)

"Many women experience psychological and social changes and stresses during menopause that they may not feel comfortable discussing with their family, including their husbands and children. It is common for family members to struggle to understand and support the psychological changes that women go through after menopause". (N, F)

The use of **social media** for promoting WFHS needs to be considered to improve the services:

"We created a Facebook page to communicate and collaborate with the community to enforce WFHS in our PHC centre". (MW, F)

"Social media can be utilised to improve services for WFHS and increase awareness". (QI, F)

"We need to use social media to provide reproductive health education to teenagers. For example, we created a WhatsApp group for follow-up and educational messages. In-person participation of young women in training activities is sometimes low". (N, F)

###### 3.1.2.1.2 Theme 1:2 healthcare provider related issues

The participants emphasised the significant influence of the healthcare provider's ownership, motivation, and belief in the value and necessity of providing comprehensive women's reproductive and family health services. One participant mentioned, *"We must ensure that healthcare providers are convinced of the increasing challenges faced by women before advising them" (D, M).* Another participant stated, *"Introducing new services is not easy. It requires full acceptance of the program to implement it effectively. We can attract a large group of clients to benefit from the program if we have a strong belief in its value" (QI, F)*.

Additional concepts relevant to **capacity building** and non-traditional training needs for healthcare providers were discussed, particularly about dealing with various disabilities:

"We need well-trained healthcare providers, especially doctors. In general, doctors are not engaged in education and awareness activities. This should be an integral part of their practice. Awareness and education efforts should be non-traditional as traditional tools are not very effective". (D, M)

"There are numerous issues that need to be tackled to enhance women's, family, and health services, particularly staff training". (MW, F)

"To enhance the services for women, family, and health, we require further training in handling women with disabilities". (N, F)

"We have received basic training for managing women with disabilities, but it is insufficient and not comprehensive. We also need training for managing women with hearing disabilities". (N, F)

###### 3.1.2.1.3 Theme 1:3 services-related issues

The next theme focused on **services.** When asked to define issues related to WHFS, participants began to express the need for additional services to be included in their centres. Some participants expressed that some services are available, but they require restructuring to cater for special women's categories:

"We would like the dietician to provide special dietary counselling programs for women with polycystic ovarian syndrome, menopausal women for weight management, and women with chronic non-communicable diseases". (D, F)

Most participants emphasised the need for additional services, especially psychological and mental health support, rehabilitation, teenage support, weight management programs for adolescents, and other health screening programs. It is important to customise certain services to meet the specific needs of women in remote served by the PHC.

“We believe that screening programs, such as those for domestic violence, breast cancer, pap smears, and osteoporosis, are crucial for the success of the WFHS program”. (N, F)

“To enhance the care provided, urgent care services need to be added to the centre to cater to women facing immediate health issues. Even though we have a pharmacy, we currently cannot dispense medications”. (QI, F)

“The availability of early screening equipment can help in attracting more women and prevent the need for referrals to distant areas, where they often fail to go. For instance, currently, we have to refer women for osteoporosis and vaginal swab screenings. We could organise group screenings and provide temporary services during campaigns. As a remote centre, it's challenging to send women to the capital or the nearest city. Remote centres are struggling”. (N, F)

“As a remote centre, we can introduce an electronic telecommunication clinic where women can be contacted by telephone or via video call for counselling or examination. In this scenario, we can collaborate with specialists in the main city and provide various services. We can also have a mobile clinic to reach people living in remote areas”. (QI, F)

One key consideration was brought out during the discussion was related to women at the menopausal age. Several services are required to be incorporated within the WFHS:

“One issue that needs special attention for women during menopause is related to urinary incontinence. Around 80-90% of menopausal women suffer from incontinence and are shy to report that. We need to introduce an education about the use of Kegel exercises to improve the condition”. (N, F)

“The provision of physiotherapist or nurse to educate women on the use of Kegel exercise will add value to the service. We need to be proactive and ask the woman if she suffer from incontinence and provide the required education”. (N, F)

Areas that are of special concern were extensively discussed among participants were related to violence and bullying:

“Violence and bullying, especially among young girls, are common in the community. We have addressed these issues with psychologists and schools’ social workers, providing rehabilitation and treatment to victims at the centre”. (D, M)

“We need to address domestic violence against women. Overcoming the challenge of introducing psychological care is a significant achievement. Building trust between the women and the doctor as well as the psychologist is a success story we are proud of”. (N, F)

Several participants expressed concerns that are related to the provision of **inclusive services:**

“We need to create an inclusive environment for women with disabilities and vulnerable women, especially older women. This area faces a significant shortage”. *(D, F)*

“Special beds are required for dealing with women with mobility disability”. (MW, F)

“For clients with hearing disability, we use an application that can translate the voice into sign language and hence communicate effectively with the women with hearing disability”. (D, M)

The use of **assigned days for services** could lead to an increase in women attendance and satisfaction with the services:

“For example, based on feedback, we decided to designate one day for young women/teenager's services and another day for menopausal women”. (QI, F)

“We assigned a doctor to a specific day so that clients could build a rapport with them”. (QI, F)

###### 3.1.2.1.4 Theme 1:4 system-related issues

The participants reached a consensus to implement **electronic medical records** in all WFHS centres. Using electronic medical records saves time, improves communication, enhances data management, and enables staff to reduce time spent searching for files, rewriting records, or correcting inaccurate information:

“We recognised the importance of automation and have contacted the electronic medical records provider to integrate clinical and operational guidelines into the system, such as electronic forms or templates for prenatal care, postpartum care, and other areas” (QI, F).

"We currently do not have an electronic medical records system at our centre, which causes difficulties in communicating with women and results in wasted time retrieving records" (N, F).

Clear clinical **guidelines and documentation** are crucial for healthcare services, as they help prevent misunderstandings and errors. They also assist project managers in tracking progress and identifying risks to keep projects on track:

“The most significant development since 2018, when we started the WFHS program, is the creation of operational clinical guidelines. These guidelines have been continuously improved and updated to align with the requirements and updates of the WFHS”. (D, F)

“One of our success stories is the teenagers’ project before marriage, focusing on family planning and pregnancy care. We have developed a clear policy to address all the related needs”. (QI, F)

“Initially, we did not have medical records for our clients when we started the WFHS program. Now, our staff has developed medical records with unified forms and templates that cater to all needs. In just one year, we have progressed from having 0% to 99% medical records”. (QI, F)

“The availability of clear standards for WFHS has allowed us to identify gaps in our practice and develop an action plan to meet the requirements”. (MW, F)

##### 3.1.2.2 Domain 2: barriers and challenges to the provision of effective WFHS

The primary themes that emerged from the HCP's focus groups regarding challenges encountered in the provision of effective WFHS were related to **healthcare providers, resources, services, culture, and challenges specific to women**.

###### 3.1.2.2.1 Theme 2:1 HCP-related barriers

The participating healthcare providers reached a consensus that staff **turnover** is a significant challenge in the healthcare sector, not only in Jordan but also globally. They emphasised that this issue presents a major challenge, as ongoing capacity building is necessary, and staff turnover makes it quite challenging.

“Staff turnover is a big challenge, particularly among doctors, nurses, and midwives”. (D, M)

"The continuous turnover of staff poses a barrier to maintaining ongoing capacity building and is an important challenge". (D, F)

“In addition to the high turnover of staff, some trained staff get transferred to another PHC after they complete their training”. (QI, F)

HCP agreed to the challenge of a **very busy** schedule and time limitations, which resulted in a high workload. This inhibited HCP to provide the most effective services to as many women as possible.

“However, I can say that the key problem is time (for the women and care providers) how can this be solved it is a big challenge”. (D, F)

“Due to the busy schedule of HCP, it is a real load on our staff to follow up on the needs of each project”. (D, M)

“The healthcare provider is so overwhelmed with tasks that they hardly find additional time for counselling or education. It is an extra burden. Therefore, counselling was limited to maternity (antenatal and postnatal) services due to the high burden on staff”. (MW, F)

Additionally, HCP reported that **change management** was one of the obstacles they faced in their daily work, particularly when several new services were added to accommodate comprehensive WFHS:

“I need to talk about the process. Every time we have something new, we face difficulties. Change management is challenging. It will take some time to implement all the new processes. (D, F)”

**Capacity building** is a crucial element for effective service delivery in healthcare, and all HCPs agreed that it poses a key challenge:

“One issue we identified during the implementation process was the need for extensive training in a busy centre”. (QI, F)

“We need to train nurses and midwives in clinical breast examination for the early detection of breast cancer and not rely on referrals for this simple task”. (N, F)

“Also, we need training on how to run projects (project management)”. (D, M)”

###### 3.1.2.2.2 Theme 2:2 limited resources

he lack of resources, both financial and human, is a critical challenge in healthcare services. All participants emphasised the importance of this issue. There has been a recent surge in the demand for healthcare services, which has exceeded the capacity of healthcare facilities. Healthcare resources are limited, while client expectations are not. The group's discussion highlighted various aspects of this challenge, particularly **the insufficient space, staff, and services:**

“We had to stop the dietician work due to a lack of financial resources” (QI, F)

“Also, not all PHCs have quality coordinators or specialists to support the implementation of WFHS standards”. (N, F)

“The most important barriers are related to resources, both human and financial” (D, M)

“Our PHC centre's main challenge is space. We need more room to provide comprehensive women's health services. Currently, we use three small rooms and a small waiting area for education. Our goal is to have separate services for young women/teenagers and older women/menopausal women”. (N. F)

“The issue of privacy was also raised, as one centre noted that all services from prenatal to postnatal and breast examinations are conducted in a single room, compromising privacy”. (MW, F)

“The shortage of staff is a key barrier to implementing WFHS in our centre effectively”. (QI, F)

“Infrastructure challenges, such as the lack of space, were also highlighted as hindrances to providing effective services, despite attempts to address it through appointment systems”. (QI, F)

###### 3.1.2.2.3 Theme 2:3 services-related barriers—lack of services

The healthcare providers highlighted the importance of service quality and availability in providing high-quality healthcare. They addressed several issues related to the services offered. The **lack of certain services** in PHC centres is discouraging for women:

“Our centre lacks an OB-GYN doctor and psychologist. We need these services to be available to encourage more women to attend. Many women do not follow through with their referrals or miss follow-up appointments”. (D, F).

“We do not have mammogram services and we hope to provide one unit to the centre as this centre is the only one in the area”. (N, F)

“The absence of dietetic clinic, hinders the successful implementation of the WFHS” (F, N)

“Even if we do not have a dietician that I strongly recommend, we need a clinic that can take care of that. We could develop protocols and clinical guidelines that can support the provision of this care”. (D, M)

“One additional thing from our experience is the need for a dietetic clinic for young girls/ teenagers. Young girls rely on Junk food a lot and that results in fast tooth decay mandating the need for dental clinic and dietitian support”. (D, F)

“Laboratory services are not available onsite, and women are often referred to labs that are not free. It was suggested that having an onsite free lab would increase the effectiveness of WFHS”., (QI, F)

**Some barriers related to services were further addressed, particularly the effectiveness of follow**-up of women after **referral** or for the provision of additional services:

“An additional challenge is related to follow-up. We reach out to women via telephone, but they often do not return for follow-up appointments, citing their busy schedules”. (QI. F)

“Postpartum follow-up is challenging as women don't return due to lack of child vaccination services within the centre. They perceive counselling as a waste of time”. (N, F)

“Referral system is there but is not effective” (N, F)

“Referral services are sometimes not accepted by women due to accessibility barriers. It is far or not within her area” (QI, F)

The introduction of **electronic medical records** in Jordan contributed to the enhancement of services and the ability to have a seamless integration of services, however, it has not been implemented in all centres. The lack of electronic medical records was perceived as a key challenge:

“We do not have electronic medical records, which makes it very slow and time-consuming”. (QI, F)

“As the medical record is based on paperwork, the woman's telephone number could be wrongly entered and hence we lose track of the woman”. (N, F)

“What needs to be done is to automate services. We do not have an electronic medical records system and hence we struggle with paperwork. We need to improve documentation”. (QI, F)

###### 3.1.2.2.4 Theme 2:4 user-related barriers

Women's involvement and collaboration are essential and influence the quality of healthcare services. Clinical outcomes depend on women's ability to provide information and cooperate with HCP. Participants argued that sometimes women contribute to ineffective service delivery:

“Since we started the work on WFHS, the problem is that women attending the services do not want to stay for further services, counselling, or education. They are usually busy with other obligations”. (N, F)

“However, I can say that the key problem is time (for the women and care providers) how can this be solved it is a big challenge”. (N, F)

“This is a case where women refuse to return for counselling sessions. In general, women resist the wait for additional group counselling services. Therefore, we provided 1:1 time-consuming counselling”. (MW, F)

“Women's counselling and training are hard to attain. Sometimes the women come to the centre and never return”. (QI, F)

“The attraction of women to other WFHS is quite challenging”. (D, F)

###### 3.1.2.2.5 Theme 2:5 cultural barriers

Norms and cultural factors influence the provision of effective services and attaining the desired clinical outcome. It affects the interaction between the HCP and women and consequently the quality of services. The provision of effective WFHS is sometimes hindered by norms and cultural barriers. Some culturally sensitive areas in healthcare services, particularly with teenagers and postmenopausal women are challenging:

"We face barriers related to customs, beliefs, and traditions in the region. Husbands do not fully accept the concept of WFHS and believe that women only attend the PHCC for treatment. They usually do not prefer counselling, especially if the counsellor is male. Additionally, they do not encourage their wives to return for postnatal follow-up visits. Our challenge lies in gaining acceptance, which is related to the norms and traditions". (D, M)

“Community culture and norms are another issue during the counselling of teenagers during attraction services through schools and community committees. It is still not accepted, there is a cultural barrier”. (N, F)

“Each geographical area presented unique challenges due to variations in culture and norms, especially when addressing the health needs of teenagers and menopausal women”. (MW, F)

“There are concerns regarding social norms, particularly in maintaining high levels of confidentiality for sensitive topics such as violence and bullying”. (QI, F)

### 3.2 Phase two cross-sectional study

Cronbach's alpha for the tool was 0.915, indicating high reliability. No questions need to be dropped for analysis. The analysis of the questionnaire was based on three sections.

#### 3.2.1 Participants characteristics

A total of 393 women attending the piloted nine centres participated in the study [118 from Royal Medical Services (RMS)] centres, 149 from Ministry of Health centres (MoH), and 126 from other non-governmental centres (NGOs). Table 1 details the baseline characteristics of the participants. The largest proportion of participants aged between 30 and 39 years (*n* = 138, 35.1%), attained higher or university degrees (*n* = 139, 35.4%), and had an acceptable level of income (*n* = 176, 44.9%). The majority of the participants lived in their designated city for more than 10 years (*n* = 308, 78.4%). Although a similar trend in the use of public transport was observed in all centre types, walking is predominant for women living near NGO centres (*n* = 42, 33.3%). Accessibility was answered by the duration required to reach the centre, where around half (*n* = 182, 46.3%) reported they required less than 15 min to reach the centre. The largest proportion of women reported that they have been using the services within the centre for more than one year (*n* = 332, 84.7%). Around 38.1% (*n* = 45) of women attending military centres whose husbands are retired, whereas government employment and hourly paid labourers are the key employment status for MoH and Gov related women (*n* = 37, 25.0% and *n* = 40, 31.7% respectively)

**Table 1:** Participants’ characteristics stratified by the used primary healthcare centre (PHC) type (Mil, military; MoH, ministry of health; Gov, governmental; *n* = number).

Demographic parameter		RMS (*n* = 118)	MoH (*n* = 149)	NGO (*n* = 126)	Total (*n* = 393)
Age [*n* (%)]	15–19	1 (0.8%)	3 (2.0%)	2 (1.6%)	6 (1.5%)
	20–29	34 (28.8%)	56 (37.6%)	28 (22.2%)	118 (30.0%)
	30–39	40 (33.9%)	39 (26.2%)	59 (46.8%)	138 (35.1%)
	40 and above	43 (36.4%)	51 (34.2%)	37 (29.4%)	131 (33.3%)
Highest level of education [*n* (%)]	No Formal Education	23 (19.5%)	8 (5.4%)	29 (23.0%)	60 (15.3%)
	Primary	17 (14.4%)	16 (10.7%)	30 (23.8%)	63 (16.0%)
	Secondary	36 (30.5%)	56 (37.6%)	31 (24.6%)	123 (31.3%)
	Higher/University	39 (33.1%)	65 (43.6%)	35 (27.8%)	139 (35.4%)
	Postgraduate	3 (2.5%)	4 (2.7%)	1 (0.8%)	8 (2.0%)
How long have you lived in this city? [*n* (%)]	Less than 4 years	2 (1.7%)	18 (12.1%)	10 (7.9%)	30 (7.6%)
	4–10 years	11 (9.3%)	20 (13.4%)	23 (18.3%)	54 (13.7%)
	Over 10 years	105 (89.0%)	110 (73.8%)	93 (73.8%)	308 (78.4%)
	Don't Know	0 (0.0%)	1 (0.7%)	0 (0.0%)	1 (0.3%)
What is the main means of transport that you use to get there? [*n* (%)]	Walk	8 (6.8%)	34 (22.8%)	42 (33.3%)	84 (21.4%)
	Public transport	43 (36.4%)	58 (38.9%)	48 (38.1%)	149 (37.9%)
	Private vehicle	67 (56.8%)	57 (38.3%)	36 (28.6%)	160 (40.7%)
How long does it take to get there from your home? [*n* (%)]	<15 min	44 (37.3%)	108 (72.5%)	30 (23.8%)	182 (46.3%)
	15–30 min	43 (36.4%)	32 (21.5%)	43 (34.1%)	118 (30.0%)
	>30 min	31 (26.3%)	9 (6.0%)	53 (42.1%)	93 (23.7%)
The employment status of the head of household? [*n* (%)]	Self Employed	8 (6.8%)	15 (10.1%)	10 (7.9%)	33 (8.4%)
	Government Employee	41 (34.7%)	37 (25.0%)	27 (21.4%)	105 (26.8%)
	Private sector	5 (4.2%)	33 (22.3%)	13 (10.3%)	51 (13.0%)
	Employer/Partner	0 (0.0%)	2 (1.4%)	0 (0.0%)	2 (0.5%)
	Hourly paid labourer	4 (3.4%)	17 (11.5%)	40 (31.7%)	61 (15.6%)
	Unpaid Family Worker	15 (12.7%)	10 (6.8%)	22 (17.5%)	47 (12.0%)
	Retired	45 (38.1%)	31 (20.9%)	10 (7.9%)	86 (21.9%)
	Other	0 (0.0%)	3 (2.0%)	4 (3.2%)	7 (1.8%)
Household income [n (%)] (Annual income in Jordanian Dinar)	Very Limited Income	9 (7.6%)	11 (7.4%)	20 (16.0%)	40 (10.2%)
	Limited Income	20 (16.9%)	41 (27.5%)	34 (27.2%)	95 (24.2%)
	Acceptable Income	61 (51.7%)	64 (43.0%)	51 (40.8%)	176 (44.9%)
	Good Income	28 (23.7%)	33 (22.1%)	20 (16.0%)	81 (20.7%)
Who lives with you? [*n* (%)]^a^	Alone	0 (0.0%)	3 (0.8%)	2 (0.5%)	5 (1.3%)
	Husband	96 (24.4%)	117 (29.8%)	105 (26.7%)	318 (80.9%)
	Children	100 (25.4%)	94 (23.9)	109 (27.7%)	303 (77.1%)
	One or more parent	10 (2.5%)	18 (4.6%)	9 (2.3%)	37 (9.4%)
	One or more parent-in-law	0 (0.0%)	1 (0.3%)	2 (0.5%)	3 (0.8%)
	Other relatives	1 (0.3%)	1 (0.3%)	2 (0.5%)	4 (1.0%)
What service did you use in the PHC? [*n* (%)]^a^	Antenatal care	56 (14.3%)	34 (8.7%)	39 (9.9%)	129 (32.9%)
	Postnatal care	66 (16.8%)	97 (24.7%)	74 (18.9%)	237 (60.5%)
	Family planning	40 (10.2%)	73 (18.6%)	61 (15.6%)	174 (44.4%)
	Postmenopausal care	5 (1.3%)	6 (1.5%)	6 (1.5%)	17 (4.3%)
	Abortion care	15 (3.8%)	1 (0.3%)	4 (1.0%)	20 (5.1%)
	Psychological services	4 (1.0%)	5 (1.3%)	51 (13.0%)	60 (15.3%)
	Adolescence care	1 (0.3%)	0 (0.0%)	5 (1.3%)	6 (1.5%)
	Nutritional support	18 (4.6%)	11 (2.8%)	25 (6.4%)	54 (13.8%)
	Dental care	92 (23.5%)	37 (9.4%)	21 (5.4%)	150 (38.3%)
	Other services	73 (18.6%)	44 (11.2%)	52 (13.3%)	169 (43.1%)
How long have you been using the services within the PHC? [*n* (%)]	<3 months	0 (0.0%)	1 (0.7%)	0 (0.0%)	1 (0.3%)
	3–6 months	0 (0.0%)	11 (7.4%)	16 (12.8%)	27 (6.9%)
	6–12 months	6 (5.1%)	19 (12.8%)	7 (5.6%)	32 (8.2%)
	>1 year	112 (94.9%)	118 (79.2%)	102 (81.6%)	332 (84.7%)

a Multiple responses.

The largest proportion of the participating women lives with their husbands and children (*n* = 318, 80.9% and *n* = 303, 77.1% respectively) and use the postnatal services (*n* = 237, 60.5%). In RMS centres the main used service is dental care (*n* = 92, 23.5%).

#### 3.2.2 Women's perception and satisfaction regarding WFHS services and facility

Women who visited the PHCCs in the last year were asked questions about the quality of women-friendly healthcare services during their visits. As reported in Table 2, the overall score of participants’ perceptions about WFHS facility and services quality was 4.07 ± 0.387 (81.4%), with the highest being for the “language spoken by the health worker was easy to understand” and “You were informed about all procedures to be done to you in a way that you understand” [4.62 (92.4%) and 4.36 (87.2%), respectively]. The least services that were perceived well by women were related to “The health worker greeted and introduced him/her to you when you arrived” and “Healthcare facilities had designated breast-feeding areas/ diaper changing area” [3.24 (64.8%) and 3.29 (65.8%), respectively].

**Table 2:** Participants’ responses regarding WFHS centre services and facility.

No.	Domain	Strongly disagree *n* (%)	Disagree *n* (%)	Neutral *n* (%)	Agree *n* (%)	Strongly Agree *n* (%)	Mean Score ± SD
1	Satisfied with waiting time in a health facility during your health services provisions	23 (5.9%)	31 (7.9%)	37 (9.4%)	175 (44.5%)	127 (32.3%)	3.9 ± 1.119
2	The healthcare centre provides you with continuous availability of basic supplies, equipment, or drugs	37 (9.4%)	57 (14.5%)	35 (8.9%)	131 (33.3%)	124 (31.6%)	3.65 ± 1.324
3	Received care from the skilled healthcare providers	7 (1.8%)	19 (4.8%)	12 (3.1%)	143 (36.4%)	196 (49.9%)	4.33 ± 0.901
4	Obtain consent or permission from you before any procedures	16 (4.1%)	20 (5.1%)	12 (3.1%)	135 (34.4%)	195 (49.6%)	4.25 ± 1.039
5	You were informed about all procedures to be done to you in a way that you understand	4 (1.0%)	23 (5.9%)	12 (3.1%)	138 (35.1%)	207 (52.7%)	4.36 ± 0.882
6	The language spoken by the health worker was easy to understand	1 (0.3%)	4 (1.0%)	5 (1.3%)	121 (30.8%)	260 (66.2%)	4.62 ± 0.594
7	The health worker greeted and introduced him/her to you when you arrived	92 (23.4%)	68 (17.3%)	13 (3.3%)	90 (22.9%)	128 (32.6%)	3.24 ± 1.614
8	The providers explained to you what is being done and what to expect during any procedures or examinations	16 (4.1%)	14 (3.6%)	15 (3.8%)	120 (30.5%)	224 (57.0%)	4.34 ± 1.007
9	Healthcare providers informed you of different options and allowed you to adopt one of your choices	26 (6.6%)	39 (9.9%)	21 (5.3%)	101 (25.7%)	173 (44.0%)	3.99 ± 1.275
10	Healthcare facilities had clean toilets	27 (6.9%)	30 (7.6%)	66 (16.8%)	80 (20.4%)	129 (32.8%)	3.77 ± 1.277
11	Healthcare facilities had adequate waiting areas	28 (7.1%)	32 (8.1%)	16 (4.1%)	136 (34.6%)	180 (45.8%)	4.04 ± 1.213
12	Healthcare facilities had designated breast-feeding areas/ diaper changing area	41 (10.4%)	27 (6.9%)	78 (19.8%)	50 (12.7%)	67 (17.0%)	3.29 ± 1.364
13	The centre is kept clean (beds, floors, windows, walls & linen)	5 (1.3%)	18 (4.6%)	22 (5.6%)	142 (36.1%)	202 (51.4%)	4.33 ± 0.877
14	The centre has clear signs to direct you to all places	7 (1.8%)	20 (5.1%)	15 (3.8%)	135 (34.4%)	207 (52.7%)	4.34 ± 0.915
15	The reception facilitates the process of my registration and initial assessment	13 (3.3%)	17 (4.3%)	16 (4.1%)	150 (38.2%)	191 (48.6%)	4.26 ± 0.973
16	Use linen, curtains or screens to ensure your privacy during examinations	13 (3.3%)	19 (4.8%)	23 (5.9%)	118 (30.0%)	207 (52.7%)	4.28 ± 1.018
17	Keep the examination room separately	19 (4.8%)	24 (6.1%)	28 (7.1%)	111 (28.2%)	202 (51.4%)	4.18 ± 1.125

Grand mean = 4.07 ± 0.387.

Table 3 summarises women's responses regarding the level of women-friendly care provision experienced by them during their visit to the centres. The sections were further subdivided into various aspects of friendly care, particularly friendly care, abuse-free care, timely care and discrimination-free care. The highest score was related to discrimination-free care (4.65 ± 0.592 (93%), and the lowest was related to timely care [3.44 ± 1.125 (68.8%)]. Overall, women's perception of how staff treated them with respect, called them by their name and spoke to them in a language they understood was highly scored among participants. Additionally, the key drawback to WFHS was related to waiting time, which scored the lowest among all WFHS-friendly care-related questions [3.16 ± 1.354 (63.2%)].

**Table 3:** Participants’ responses regarding WFHS centre respect and dignity.

No.		Strongly disagree *n* (%)	Disagree *n* (%)	Neutral *n* (%)	Agree *n* (%)	Strongly Agree *n* (%)	Mean ± SD
A.	Friendly care				4.49 ± 0.653		
1.	I felt that the healthcare providers cared for me with a kind approach	8 (2.0%)	12 (3.1%)	22 (5.6%)	101 (25.7%)	250 (63.6%)	4.46 ± 0.889
2.	The healthcare providers treated me in a friendly manner	8 (2.0%)	14 (3.6%)	25 (6.4%)	97 (24.7%)	249 (63.4%)	4.44 ± 0.913
3.	The healthcare providers were talking positively about pain and relief	7 (1.8%)	9 (2.3%)	15 (3.9%)	120 (30.9%)	237 (61.1%)	4.47 ± 0.827
4.	The healthcare provider showed his/her concern and empathy	10 (2.6%)	16 (4.1%)	27 (6.9%)	119 (30.4%)	219 (56.0%)	4.33 ± 0.956
5.	All healthcare providers treated me with respect as an individual	5 (1.3%)	8 (2.0%)	9 (2.3%)	129 (33.0%)	240 (61.4%)	4.51 ± 0.757
6.	The healthcare providers speak to me in a language that I can understand	0 (0.0%)	5 (1.3%)	3 (0.8%)	117 (31.0%)	253 (66.9%)	4.63 ± 0.573
7.	The healthcare provider called me by my name	2 (0.5%)	7 (1.8%)	3 (0.8%)	123 (31.4%)	257 (65.6%)	4.6 ± 0.652
B.	Abuse Free care				4.31 ± 0.653		
8.	The healthcare providers responded to my needs whether or not I asked	8 (2.2%)	24 (6.6%)	29 (8.0%)	135 (37.2%)	167 (46.0%)	4.18 ± 0.986
9.	The healthcare providers shouted at me because I hadn't done what I was told to do- NEGATIVE	297 (75.8%)	67 (17.1%)	6 (1.5%)	16 (4.1%)	6 (1.5%)	4.61 ± 0.835
10.	I was allowed to practice cultural rituals in the facility	3 (1.3%)	16 (7.0%)	93 (41.0%)	52 (22.9%)	63 (27.8%)	3.69 ± 0.997
11.	The healthcare providers encouraged you to ask questions and respond with politeness and truthfulness	7 (1.8%)	21 (5.4%)	10 (2.6%)	147 (38.0%)	202 (52.2%)	4.33 ± 0.905
C.	Timely care				3.44 ± 1.125		
12.	I was kept waiting for a long time before receiving service-(NEGATIVE)	78 (20.1%)	100 (25.8%)	71 (18.3%)	83 (21.4%)	56 (14.4%)	3.16 ± 1.354
13.	Service provision is always delayed due to the health facilities’ internal problem- (NEGATIVE)	135 (36.3%)	106 (28.5%)	60 (16.1%)	51 (13.7%)	20 (5.4%)	3.77 ± 1.227
D.	Discrimination free care				4.65 ± 0.592		
14.	Some of the healthcare providers do not treat me well because of some personal attribute- (NEGATIVE_	271 (72.1%)	82 (21.8%)	10 (2.7%)	11 (2.9%)	2 (0.5%)	4.62 ± 0.728
15.	Some healthcare providers insulted me and my companions due to my personal attributes-(NEGATIVE)	278 (73.7%)	84 (22.3%)	7 (1.9%)	7 (1.9%)	1 (0.3%)	4.67 ± 0.633
16.	Healthcare providers respect you regardless of your cultural norms, religion, and beliefs throughout your stay in this health facility	3 (0.8%)	6 (1.5%)	9 (2.3%)	99 (25.3%)	274 (70.1%)	4.62 ± 0.683

Grand mean = 4.32 ± 0.426.

Table 4 presents the participating women's demographic data and the mean scores for the two main dimensions. The analysis for each dimension of WFHS showed significant differences between the perceived quality of WFHS with respect and dignity dimension and age. Middle-aged women (30–39 years) were more satisfied with the services when compared with older and younger women (*p* = 0.02). Women with no or low levels of education were more satisfied with WFHS services and facilities, and respect and dignity dimensions (*p* < 0.001 and 0.003, respectively), and the associations between perceived quality and the time required to get to the centre were significant (*p *< 0.001). Concerning the duration of the use of services, there was no significant difference in women's satisfaction with the services and duration of their use (*p* > 0.5).

**Table 4:** Stratification of participants’ mean satisfaction scores regarding the WFHS centre according to women's age, educational level, duration of service use and distance from home.

Domain	N	Mean score ± SD	
		Services and Facility	Respect and Dignity
Age			
15–19	6.00	3.85 ± 0.34	4.08 ± 0.62
20–29	118	4 ± 0.7	4.23 ± 0.66
30–39	138	4.2 ± 0.6	4.45 ± 0.55
≥40	131	4.07 ± 0.67	4.34 ± 0.54
Total	393	4.09 ± 0.65	4.34 ± 0.59
P (ANOVA)		0.075	0.02
Educational level			
None	60	4.43 ± 0.56	4.6 ± 0.4
Primary	63	4.1 ± 0.52	4.36 ± 0.44
Secondary	123	3.99 ± 0.69	4.26 ± 0.65
Higher/University	139	4.04 ± 0.68	4.31 ± 0.64
Postgraduate	8	3.8 ± 0.57	4.12 ± 0.34
Total	393	4.09 ± 0.65	4.34 ± 0.59
P (ANOVA)		<0.001	0.003
How long does it take to get there from your home?			
<15 min	182	3.9 ± 0.7	4.18 ± 0.65
15–30 min	118	4.22 ± 0.61	4.42 ± 0.53
>30 min	93	4.29 ± 0.52	4.56 ± 0.42
Total	393	4.09 ± 0.65	4.34 ± 0.59
P (ANOVA)		<0.001	<0.001
How long have you been using the services within the centre?			
<3 months	1	3.88	3.69
3–6 months	28	4.03 ± 0.42	4.25 ± 0.45
6–12 months	32	3.93 ± 0.58	4.27 ± 0.63
>1 year	332	4.11 ± 0.68	4.36 ± 0.59
Total	393	4.09 ± 0.66	4.34 ± 0.59
P (ANOVA)		0.449	0.440

To further understand the correlation among WFHS domains, the Spearman correlation coefficients were determined (Table 5). The results showed that there was a statistically significant correlation among all WFHS domains, with Spearman's coefficient values exceeding 0.3 in all domains, indicating a high level of correlation within the tool.

**Table 5:** Correlation analysis for participants’ responses according to the WFHS survey domains.

Spearman's			Correlations					
			Service quality	Friendly care	Timely care	Discrimination free care	Services satisfaction	Abuse free care
1.	Service Quality	Correlation Coefficient	1.000	.766**	.432**	.522**	.997**	.636**
		Sig. (2-tailed)		<.001	<.001	<.001	<.001	<.001
2.	Friendly Care	Correlation Coefficient	.766**	1.000	.408**	.602**	.766**	.810**
		Sig. (2-tailed)	<.001		<.001	<.001	<.001	<.001
3.	Timely Care	Correlation Coefficient	.432**	.408**	1.000	.180**	.431**	.372**
		Sig. (2-tailed)	<.001	<.001		.009	<.001	<.001
4.	Discrimination Free Care	Correlation Coefficient	.522**	.602**	.180**	1.000	.521**	.658**
		Sig. (2-tailed)	<.001	<.001	.009		<.001	<.001
5.	Services Satisfaction	Correlation Coefficient	.997**	.766**	.431**	.521**	1.000	.632**
		Sig. (2-tailed)	<.001	<.001	<.001	<.001		<.001
6.	Abuse Free Care	Correlation Coefficient	.636**	.810**	.372**	.658**	.632**	1.000
		Sig. (2-tailed)	<.001	<.001	<.001	<.001	<.001	

** Correlation is significant at the 0.01 level (2-tailed).

## 4 Discussion

In this study, we explored the views of users and healthcare providers on the implementation of women-friendly health services through a combination of qualitative and quantitative research methods. Our main aim was to understand the experience of healthcare providers and women with regard to the newly introduced comprehensive WFHS in Jordan, and determine the key challenges encountered in the provision of such services. Further, we wanted to explore the sociodemographic factors associated with women's satisfaction with WFHS.

It is important to address the health and well-being of women to achieve health equity for all. Women comprise 67% of the global health and social workforce (31), so when women are healthy, it benefits entire communities, especially children and young people. Therefore, it is valuable to provide health services that cater to women's health across their lifespan (32).

In Jordan, a total of 673 health centres are distributed throughout the country, with 109 of them being comprehensive (33). The centres serve more than 11.4 million people, with 50.1% being women (34). In 2017, there were 10,676,838 visits to these centres, with an average of 16,000 visits per centre per year (33). The Ministry of Health has adopted a strategy to improve utilisation and reduce the burden on secondary health services by expanding health insurance coverage, implementing family medicine programs to integrate more services in PHC, particularly noncommunicable disease, and training general practitioners on PHC and family medicine aspects. According to this study in the piloted centres, primary health services are highly utilised, particularly among women with low to average income (79.3% of participants). Therefore, increasing health insurance coverage is expected to boost the utilisation of services. It was reported that PHC services utilisation was positively associated with low-income individuals in Jordan (35).

This study has identified multiple challenges HCPs encounter in their pursuit to implement WFHS in their centres. These challenges include a high turnover of staff, busy HCP, limited resources, the need for continuous training, lack of certain services and cultural constraints. Often, women resist change due to hectic lives. These findings are consistent with systematic review and meta-analysis studies (36, 37), which have noted financial resources as a key challenge in providing essential health benefits for clients in low and middle-income countries. These results also align with our findings regarding women's perceptions of the services, indicating that women's satisfaction regarding the availability of basic supplies, equipment, or drugs was below average (3.65 ± 1.324). Ultimately, limited resources led to poor satisfaction with physical environment-related issues, particularly the availability of breastfeeding areas, counselling rooms, and toilet cleanliness. Due to the very busy schedule of healthcare providers, women reported that the health workers did not greet or introduce themselves upon their arrival. Such findings are in concordance with the findings from other qualitative and quantitative studies in Jordan (35, 38, 39).

The provision of integrated services at the point of delivery has been found to improve coordination and service delivery by offering services together. Moreover, integration ensures that services are managed and delivered together, resulting in efficient and high-quality service that enhances public access and ultimately improves health (40). That specific issue was consistently raised by the HCP participating in the study. One qualitative research finding was that HCP reported the need for additional health services to be added to the centres to enhance WFHS or added through community integration and awareness programs. Various research reported that integration can occur exclusively at the facility level or both the facility level and in the community. Community health workers raise awareness, provide information, and conduct household visits to identify women in need and deliver required services (41). Some studies reported the need for integrating noncommunicable disease management in LMIC in PHC (42, 43). A scoping review of 111 research articles by Dodd et al., emphasised the importance of effective and institutionalised community integration and engagement in improving service demand, which aligns with our findings (44). Our study reported the need for more community integration and awareness programs for effective WFHS.

The concerns raised by healthcare providers (HCP) were related to the need for inclusive services, which require resources and capacity building of staff to effectively serve women with disabilities. These concerns are consistent with previous global studies, which have shown that inaccessible facilities, limited resources, and a shortage of trained healthcare providers are common barriers to quality healthcare services for clients with various disabilities (45–47). Therefore, it is important to develop strategies to augment the financial and human resources levels to ensure equitable access to PHC services for women with disabilities. WHO has recommended training community health workers as a way to improve access to PHC services for individuals with disabilities (48).

Quantitative research found that women used mainly antenatal (32.9%), postnatal care (60.5%), family planning (44.4%) and dental care (38.3%). Further, the new services related to psychology, postmenopausal and adolescent care were fairly utilised due to the recent addition of such services. Based on the sample in the present study, it can be concluded that the awareness was not sufficiently applied in PHC centres to capture more utilisation of other services.

Another finding from the quantitative study was related to women-friendly care that some women held the view that HCPs do not pay attention to their needs, and they receive little empathy with long waiting times. The patients were eager to get much attention from the HCPs, which is consistent with earlier research results. A study by Vinson and Underman reported that clients in healthcare progressively wanted to interact emotionally with HCPs (49). Furthermore, a scoping review identified that there is limited compassion, respect and care service provision in healthcare. Several studies reported that women do not want to pursue care from healthcare providers who mistreat and abuse their dignity (50). Lack of training, high workload, and limited resources were found to be the main reasons, which is in line with our qualitative results. Therefore, healthcare management should consider compassion, respect and care in healthcare service delivery by employing on-the-job training, monitoring and evaluation, and performance appraisal (51). HCPs should listen actively to understand women's concerns about their condition, address those concerns, and help set reasonable expectations to establish a harmonious HCP-client relationship.

The finding that some women perceived limited attention and empathy from healthcare providers stresses the fundamental role of user–provider communication in WFHS. Effective communication is regularly correlated with improved satisfaction, treatment adherence, and reduced health inequalities. However, physician trust continues to be moderate, with only 58% of adults in the United States expressing confidence in physicians (52). Studies have shown that proper engagement, active listening, and clear information-sharing are critical elements of service utilisation and perceived healthcare quality (53). Therefore, beyond structural improvements, WFHS implementation should prioritise communication skills training, supportive supervision, and workload management to ensure that providers can deliver empathetic and responsive care.

Ensuring respectful care remains a concern for women seeking healthcare services, especially in Low- or Middle-Income Country (MLIC), as it impacts the quality of care provided (54–58). Women-friendly and respectful care encompasses several aspects, which were examined in the quantitative section of the study. In research conducted by Mdoe and colleagues, two main components of women-friendly care were identified: empathy, which includes a friendly welcome, considerate language, timely care delivery, and sharing of information; and an enabling environment, characterised by a well-maintained physical space that respects privacy and contains all necessary equipment and supplies (59). Our findings suggest that healthcare services in primary healthcare centres in Jordan were generally perceived as women-friendly, with an overall score of 4.32 ± 0.426 (86.4%). Meeting women's expectations can positively influence healthcare utilisation and may serve as an indicator of high-quality care (60). To better meet women's expectations, healthcare providers should engage in discussions with women about their treatment plans, including short- and long-term goals, to foster realistic expectations (61).

To understand the determinant factors associated with women's satisfaction with women-friendly services regression analysis was conducted. Interestingly, satisfaction increased with age till 39 years and then declined after that. Young women were the least perceiving of women-friendly care. Similar results were reported by Jaipaul and Rosenthal where satisfaction increased with age but declined after the age of 65 years (62). Several research studies reported that patient satisfaction with health had a positive association with age (63–65).

The education level of women impacted satisfaction, where satisfaction was higher in those who did not receive formal education or those with primary and secondary education. Women with postgraduate education demonstrated the lowest satisfaction with both sections of the survey (satisfaction with the facility and services as well as satisfaction with respect and dignity, *p*-value <0.001 and 0.003, respectively). A similar trend was reported (66, 67). A study in a Middle Eastern country (Saudi Arabia) reported similar findings where patients with lower education levels were more satisfied with primary healthcare services in Riyadh (68). Further, higher levels of education can render people more ambitious, resulting in higher expectations that are further difficult to fulfil, which could reduce their levels of satisfaction (69). Although higher satisfaction among less educated women may appear positive, it does not necessarily imply advanced quality of care. Lower expectations or reduced awareness of service standards may impact reported satisfaction. Therefore, satisfaction scores should be interpreted carefully when reviewing equity and quality.

Travelling distance to healthcare centres was reported to impact client satisfaction with healthcare services (67). The findings of this study also revealed that travelling distance to the PHC centre was also found to be a significant determinant factor associated with women's satisfaction with WFHS. Women requiring less than 30 min to reach the centre were less satisfied with the service than those requiring a longer duration to reach the centre. It seems that the time to travel was not an issue for women utilising the services within the centres. That is inconsistent with various findings that time is a salient barrier to client satisfaction (70). However, it might be that those who took longer use their vehicles or public transportation, which could be convenient. A study by Syed et al. reported a positive association between convenient transportation and the utilisation of PHC services (71). Also, this could be attributed to discrepancies between the centres’ locations. Those living in rural areas might require time to reach the centres.

Overall, these findings suggest that effective WFHS implementation needs more than service availability; it requires systemic investment in workforce capability, infrastructure, communication training, and equity-intensive monitoring. Policymakers need to adopt a phased, resource-sensitive strategy that prioritises both structural readiness and respectful, user-centred healthcare to ensure balanced improvements in women's health outcomes.

### 4.1 Strength and limitations

This research conducted the first exploration in Jordan of the perspectives of users and providers of WFHS. There is a limited number of publications in LMIC assessing the effectiveness of the WFHS program. The study used both qualitative and quantitative methods to offer comprehensive information and bridge a gap in research on the use of integrated services for women in the WFHS program. The research included a diverse and extensive sample of healthcare providers and women. Additionally, it investigated the necessary improvements to advance the WFHS in Jordan as a representative of LMICs. Consequently, the study provides scientific evidence for measures to enhance and expand the implementation of WFHS in healthcare. The study is useful for policymakers to understand the status of WFHS in piloted centres and help plan for the expansion process.

This study has some limitations. Firstly, the study in Phase 2 used self-reported data, which may be subject to response bias; this was mitigated by ensuring anonymity, voluntary participation, and clear instructions to encourage accurate and honest responses from users. Secondly, there is a limited number of studies that reported the impact of integrated WFHS, particularly in LMIC, which limited our ability to compare our findings with similar programs; we addressed this by drawing on broader evidence from related women-centred healthcare services and contextualising our findings accordingly. Owing to the study design (questionnaire with closed-end answers for phase 2), we might have missed some information that could have been captured using open-ended questions; this was partially addressed by including Phase 1 qualitative focus discussion groups to explore a deeper insight into the services and complement the survey data. Finally, healthcare providers in the qualitative phase were recruited using a snowball approach, which may have introduced selection bias and limited diversity of perspectives; however, participants were selected based on their direct contribution in WFHS implementation to ensure relevance.

## 5 Conclusions

The integration of health services for women through the WFHS program in Jordan was well received by users and providers, which is expected to strengthen primary healthcare services. However, significant implementation challenges were identified, including staff turnover, heavy workload, limited financial resources, the need for continuous professional development, service gaps, and cultural constraints. These challenges were reflected in users’ moderate satisfaction levels, particularly regarding availability of supplies, physical infrastructure (e.g., breastfeeding areas, counselling rooms, toilet cleanliness), and healthcare provider–user communication. Likewise, additional services need to be integrated into the PHCC. These include noncommunicable disease management, dietitian services, psychological support, and improved inclusive services for women with disabilities. This study was conducted one year after the implementation of WFHS standards, making it the right time for an early evaluation of the services to enable early intervention. To enhance the WFHS program's impact, policymakers and PHC leaders need to prioritise resource allocation, infrastructure improvement, continuous staff training, workload management, and expansion of the recommended integrated services. Addressing these areas through phased, resource-sensitive strategies can strengthen WFHS sustainability in Jordan and inform similar initiatives in other LMICs.

## Data Availability

The original contributions presented in the study are included in the article/Supplementary Material, further inquiries can be directed to the corresponding author.
