# Supplementary material for: An insight into the experience of healthcare providers and users of women-friendly health services in Jordan—a mixed method study
Source: Front Glob Womens Health. 2026 Apr 8;7:1772412. doi: 10.3389/fgwh.2026.1772412 (PMC13099807; doi:10.3389/fgwh.2026.1772412)
Supplement: Supplementary file 1 [file Table1.docx]

**Table S1:** Thematic analysis of healthcare providers' experiences in WFHS addressing two domains

| Domain / Theme | Subthemes |
| --- | --- |
| 1. **Healthcare issues for women that should be incorporated into the WFHS to improve the service?** | |
| - 1. Theme) Community Integration and Communication | - Awareness programs - Integration with community services - Family Support - Social media |
| - 1. (Theme) HCP Related Issues | - Ownership from HCP - Capacity Building |
| - 1. (Theme) Services Related Issues | - Additional Services - Inclusive services - Assigned days or providers for specific services |
| - 1. (Theme) System Related Issues | - Medical Records - Documentation and clinical guidelines |
| 1. **Barriers and challenges in WFHS** | |
| - 1. (Theme) HCP Related Barriers | - Staff Turnover - Busy HCP - Change management. - Capacity Building |
| - 1. (Theme) Limited Resources | - Limited Space - Resources Limited (Human and Financial) |
| - 1. (Theme) Services Related Barriers | - Ineffective follow-up - Ineffective referral - Lack of Services - Medical records |
| - 1. (Theme) Women-Related Barriers |  |
| - 1. (Theme) Cultural Barriers |  |
